# When Passion Does Not Change, but Emotions Do: Testing a Social Media Intervention Related to Exercise Activity Engagement

**DOI:** 10.3389/fpsyg.2020.00071

**Published:** 2020-02-05

**Authors:** Silje Berg, Jacques Forest, Frode Stenseng

**Affiliations:** ^1^Department of Psychology, Norwegian University of Science and Technology (NTNU), Trondheim, Norway; ^2^Department of Organization and Human Resources, Université du Québec à Montréal (UQAM), Montreal, QC, Canada; ^3^Department of Education and Lifelong Learning, NTNU, Trondheim, Norway; ^4^Queen Maud University College, Trondheim, Norway

**Keywords:** social media, exercise, intervention, self-determination theory, dualistic model of passion

## Abstract

Grounded in self-determination theory and the dualistic model of passion, the present study tested whether a social media intervention could promote harmonious passion and positive emotions related to exercise activities. A 4-week intervention managed through an Instagram™ account was designed to promote more harmonious passion and less obsessive passion, as well as more positive emotions and less negative emotions related to participants’ favorite exercise activities. A web-based questionnaire was distributed to 518 young adults (mean age 26.5) before and after the intervention. Participants were randomly assigned to an intervention group (*N* = 226) or control group (*N* = 292). The intervention did not predict change in passion for the activity. However, the intervention predicted more positive emotional outcomes from the activity, statistically controlled for emotions reported at baseline and negative emotions reported at follow-up. Results indicate that digital platforms may be relevant in promoting more physical activity in the population, through the facilitation of more positive emotions related to exercise.

## Introduction

The World Health Organization (2010) recommends that adults should engage in more than 150 min of physical activity of moderate intensity (e.g., walking briskly) per week, or at least 75 min of vigorous physical activity (e.g., jogging, playing tennis, or soccer). However, one in four adults are too inactive held up to these standards (World Health Organization, 2010). Given the imperative role of regular physical activity in maintaining health and well-being in the community, health authorities strive to find approaches that motivate people to engage in regular exercise. However, due to new ways to communicate (e.g., through social medias such as Instagram, Facebook, and Snapchat), the range of potential tools targeted at motivating individuals to become more physically active has broadened. Still, relatively few studies have empirically investigated the potential effect of using social media as a way to promote more and better motivation toward physical activity (for a review, see Welch et al., 2018). Thus, the aim of the present study was to test the effect of a social media intervention targeted at promoting more adaptive motivation and positive emotions toward physical exercise. The content of the intervention was developed from principles implemented in self-determination theory (SDT; Deci and Ryan, 1985, 2000) and the dualistic model of passion (DMP; Vallerand et al., 2003; Vallerand, 2015).

### Motivation Toward Physical Activity Engagement

Self-determination theory (Deci and Ryan, 1985, 2000) is a universal and encompassing theory of human motivation that is applicable to sport and exercise activities. A meta-analysis including nearly 200 datasets supports the relevance of SDT for a wide range of health outcomes (Ng et al., 2012). By asserting the quality of motivation, SDT draws a distinction between intrinsic and extrinsic forms of motivation. For example, a person performing a particular behavior for external reasons, such as money or reputation, is directed by external regulation at the expense of volitional—or self-determined—motives for the behavior. Intrinsic motivation, on the other hand, reflects engagement in the behavior motivated from satisfaction of performing the behavior itself. When engagement is rooted in such autonomous motives, there are no or few external contingencies attached to the activity (see e.g., Hagger and Chatzisarantis, 2007). Notably, rather than viewing extrinsic and intrinsic motivation as opposites, SDT identifies several levels of quality of motivation that are considered as being more, or less, self-determined, ranging from compliance to rules utterly defined by others (external regulation), through external regulation which is more integrated in the self (identified regulation), to purely self-driven interest (intrinsic regulation). When behavior is self-determined and not externally regulated, it will more likely contribute to the satisfaction of three basic psychological needs (Deci and Ryan, 2000): *autonomy* (the desire to exert behavior in concordance with one’s true values and aspirations), *competence* (a sense of accomplishment and development), and *relatedness* (the need to feel appreciated by people one feels connected to).

One branch of research within the SDT paradigm has focused on motivation for sport and exercise activities. According to Ryan and Deci (2007, 2017), adaptive motivation in sport and exercise activities is most likely to occur when individuals partake in the activity for autonomous reasons. More self-determined forms of exercise motivation are related to greater psychological well-being (Wilson and Rodgers, 2007), more exercise engagement and adherence over time (Chatzisarantis et al., 2003), higher perceived competence (Goudas et al., 1994), better quality of life (Gillison et al., 2006), and increased self-efficacy to overcome barriers to regular exercise (Thøgersen-Ntoumani and Ntoumanis, 2006). Moreover, it has been shown that (general) autonomous motivation predicts life longevity (Weinstein et al., 2019).

### The Dualistic Model of Passion

The dualistic model of passion (DMP; Vallerand et al., 2003; Vallerand, 2015), which is partly derived from self-determination theory, uses the term “passion” to describe the intense motivation for activities that people love, value, and invest much time and energy in. What the dualistic model of passion adds beyond self-determination theory (Deci and Ryan, 1985, 2000) is a more specific focus on a particular activity, in the sense that it represents a long-term passion for the person, and that motivation for this activity is internalized in the person’s identity in a controlled or autonomous fashion based on individual characteristics and/or interpersonal processes. The passionate activity becomes self-defining, so that a person interested in in running will describe him or herself as a “runner” for example, not just a person who likes to run, and thus has internalized the activity as a central feature of one’s identity (Vallerand et al., 2003). Furthermore, the DMP proposes that passion can be integrated into one’s self through different regulatory processes, depending on how the passionate activity has been internalized into one’s identity. This internalization of the activity gives rise to two qualitatively distinct forms of passion, namely *harmonious passion* and *obsessive passion*, each of which leads to contrary psychological outcomes. Harmonious passion, on the one hand, is characterized by a balanced and flexible involvement in the passionate activity. The activity occupies a significant place in the person’s daily life but remains in harmony with other aspects in life, causing no or little conflict. It is purported that harmonious passion originates from an autonomous internalization of the activity in the person’s identity: the passionate activity has been freely chosen and personally endorsed (Vallerand et al., 2003). Thus, the behavior toward the activity is regulated by motivational processes indicative of volitional functioning. With harmonious passion, the person feels in control over the activity and can thus fully partake in the activity with a sense of volition and flexibility (Vallerand et al., 2003). Consequently, such balanced and secure engagement in a passionate activity is related to positive experiences and affect, which are conducive to higher level of happiness, satisfaction, and enjoyment (Vallerand, 2015).

Obsessive passion, on the other hand, is also characterized by intense love and strong commitment for an activity, but the interest has a downside. The commitment related to the activity threatens one’s self-control, as the person experiences an obsessive need to do the activity, often at the expense of other important aspects in life. Obsessive passion is claimed to originate from controlled internalization of the activity: the passionate activity has been chosen and personally endorsed, but also affected by intrapersonal and/or interpersonal pressure by either contingencies such as feelings of social acceptance or self-esteem, or because of the uncontrollable excitement derived from activity engagement (Vallerand et al., 2003). Being obsessively passionate, the person cannot help but engage in the passionate activity, even when negative outcomes are inevitable. As illustrated in a study by Rip et al. (2006), rigid persistence leads obsessively passionate dancers to continue dancing when injured, leading to chronic injury instead of healing.

Harmonious and obsessive passion has been shown to be rather stable over time (Vallerand et al., 2003; Lalande et al., 2017). However, since passion is—at its fundamental core—a motivational construct, one may expect it to be moldable. At present, few studies have addressed this question. However, there are some indications that passion may be affected through external sources, but perhaps foremost during the internalization of the activity. For example, Mageau et al. (2009) found that autonomy support from a parent or a significant adult (e.g., a coach or a teacher) predicted more harmonious passion among children and teenagers. Also relevant, although conducted in a work setting, Forest et al. (2012) found that an intervention program targeted at promoting the use of signature strengths in the organization predicted an increase in harmonious passion among employees over at 2-month period. To our knowledge, no interventions have been developed—or empirically tested—that are targeted toward amending levels of harmonious and obsessive passion in the exercise setting.

### Motivation for Exercise and Emotional Outcomes

Several studies indicate that people who report enjoyment, happiness, and satisfaction in exercise settings participate more regularly, and for longer periods (Steptoe and Butler, 1996; Amnesi, 2010). As with many health behaviors, physical activity is highly dependent upon effective self-regulation, and research has shown that affective responses to an activity may be a key component of exercise regulation (Kwan and Bryan, 2010). In brief, autonomous motivation has been found to be a predictor of positive emotions outcomes, and satisfaction with exercise (Blanchard et al., 2009), greater psychological well-being (Sebire et al., 2009), and enhanced perceptions of physical self-esteem or self-worth (Thøgersen-Ntoumani and Ntoumanis, 2006; Sebire et al., 2009).

Studies also indicate a link between type of passion and affective outcomes. For example, individuals with a more harmoniously passionate engagement are conducive to higher enjoyment, satisfaction, and happiness. On the contrary, individuals with more obsessive passion are conducive to lower enjoyment, satisfaction, and happiness (Vallerand et al., 2003). Harmonious passion correlates with more positive emotions in activity engagement (Vallerand et al., 2003; Stenseng et al., 2015) and higher life satisfaction (Vallerand et al., 2003). On the other hand, obsessive passion is associated with negative outcomes such as negative emotions like shame and guilt (Vallerand et al., 2003), but also with higher risk for burnout (Schellenberg et al., 2013), lower self-esteem (Stenseng and Dalskau, 2010), and more intrapersonal conflict (Stenseng et al., 2011). In sum, several studies indicate that obsessive passion as a predominant type of passion for an activity impedes the experience of positive emotions, whereas harmonious passion contributes to more positive emotional outcomes in relation to the activity.

### Social Media Interventions and Motivation for Exercise

Developing effective interventions is a crucial part of promoting good health. As such, social media offers an opportunity to reach many individuals at low cost. On Facebook, Instagram, Snapchat (etc.), it is possible to communicate with high frequency to the target group through text, images, and clips. Two-way communication is also available, and communication among followers may also be facilitated. In a systematic review on social media interventions for diet and exercise behaviors, Williams et al. (2014) pinpointed several advantages of this form of interventions, but they did not find overall evidence that such interventions significantly improve the targeted behavior. However, in contrast, more recent studies have shown that social media interventions can improve smoking cessation (Naslund et al., 2017a,b), increase time spent on physical activity engagement (Zhang et al., 2015), and promote improved fitness among psychiatric patients (Naslund et al., 2017a,b). In other words, social media interventions may affect health behavior, but few interventions have addressed the motivational underpinnings that contribute to the change of behavior. Limited knowledge on how social media interventions affect behavior through changes in motivation may be one of the reasons why such efforts fail to have the anticipated effect. As such, the present study was aimed at revealing some of the “core components” (Heath et al., 2012) in social media intervention targeted at changing behavior.

### The Present Study

The present study tested whether a social media intervention could modify participants’ passion and affective outcomes in relation to an exercise activity. In a longitudinal design, with pre- and post-tests and a control group, an intervention was carried out through an Instagram account, where tailored material in form of text, links, and images was posted every third day over a 4-week period. Based on self-determination theory (Deci and Ryan, 1985, 2000) and the dualistic model of passion (Vallerand et al., 2003; Vallerand, 2015), information was published to promote autonomy and awareness of basic need satisfaction through the activity, including stimulation of self-awareness concerning the participants’ own predominant passion, which was proposed to promote more adaptive passion and better affective outcomes from activity engagement. It was hypothesized that participants in the intervention group—relative to the control group—would strengthen their harmonious passion toward the exercise activity, whereas obsessive passion would attenuate in the intervention period. Also, in the intervention group, we hypothesized that positive emotions would increase, whereas negative emotions would decrease during the intervention period. Questions about the grade of adherence to the intervention were also included in the study.

## Method

### Participants

**Figure 1:**
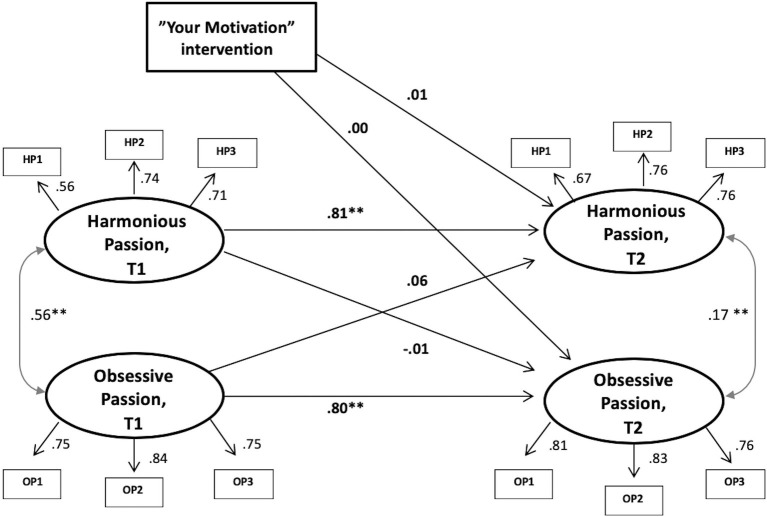
Structural equation modeling of the results. The two passion constructs and the intervention variable. Note: **p < 0.01; Intervention variable: Intervention group = 1, Control group = 0.

A total of 518 individuals participated in the study. In the intervention group, 226 participants (217 women, 9 men) completed the Time 1 questionnaire, while 121 completed the Time 2 questionnaire (53.5% response rate). In this group, the mean age of participants was 27 years (SD = 6.68), and the average time spent on exercise per week was 3.5 h (SD = 0.89). Participants who answered “No” whether they had participated in the intervention or not (*N* = 8) by following the Instagram account “DinMotivasjon” (in English: “YourMotivation”) were excluded from further data analyses. In the control group, 292 participants (232 women, 60 men) completed the Time 1 questionnaire, while 163 completed the Time 2 questionnaire (55.8% response rate, see Figure 1). The mean age of participants in the control group was 26 years (SD = 5.81), and the average time spent on physical activity per week was 3.7 h (SD = 0.93). Participants in both groups had been participating in their favorite exercise activity for an average of 2.5 years (SD = 0.50), reporting strength training (38.5%), cardio/running (20.6%), and aerobic classes (13.4%) as their favorite activity.

### Procedures

Participants were recruited through Facebook, Instagram, and a student webpage. Some participants were also recruited through the blog www.fitnok.no run by a personal trainer. Inclusion criteria were: (1) regular physical activity engagement and (2) access to Instagram though smartphone or tablet. Participants were randomly assigned to either the control or the intervention group after they had shown interest for the study. Participants who were assigned to the control group were told that they would be contacted again by email to complete a follow-up questionnaire in about 4-week time. They were also told that they could partake in the intervention after they had completed the follow-up questionnaire.

All participants completed a web-based questionnaire^1^ in which they reported socio-demographics, exercise habits, passion, and affect. Following the completion of the questionnaire, participants in the intervention group were instructed to follow the unique Instagram profile “Dinmotivasjon,” or “YourMotivation” in English (only available for participants in this group). At follow up, the questionnaire had identical content as at baseline, except for questions regarding participants’ rate of involvement and adherence in the intervention (in the intervention group). When following the interventions’ Instagram profile, posts presented by the researchers would emerge automatically on each participant’s Instagram-stream, with an immediate notification on their mobile phone, iPad, or tablet.

All participants were informed that participation in the study was voluntary. Participants were told that the project was about motivation and exercise, and that their answers would be anonymous. To clarify the participants’ consent, each participant was given the option of ticking either “yes” or “no” for consent in the questionnaire. In order to contact the participants for the follow-up, the project requested the participants’ email addresses. Beforehand, the study had been approved by the Regional Committee for Research Ethics, Mid-Norway^2^.

### The YourMotivation-Intervention

Intervention posts were published on this Instagram profile every third day for the following 4 weeks (see Appendix). The intervention material was grounded in SDT-research (Ryan and Deci, 2006) and theory about passion (Vallerand et al., 2003), with the aim of providing support, knowledge, and awareness based on the postulates of the theories. At startup, the Instagram profile provided introductory knowledge to the participants. The goal was to make participants reflect over the motivational aspect of their activity engagement, e.g., interpret their motivation in light of: (1) satisfaction of basic psychological needs, (2) harmonious and obsessive passion, (3) and their emotions related to the activity. In the following posts, we primarily presented material on SDT-principles, what passion is, and how to regulate engagement in exercise in a better manner. Here is an excerpt of one of the posts, translated from Norwegian:

Do you exercise because you really want to, or because you feel you have to? Your answer is related to the type of passion you have for your exercise activity. There is a distinction between two types of passion. Harmonious passion is defined as something you can spend time and energy on, without experiencing conflicts with other parts of your life. You feel in control of your need to exercise, even when it is a big part of your life. Obsessive passion, on the other hand, is also characterized by strong commitment to the exercise, but nevertheless, this strong commitment may develop beyond your own control so that your interest for the activity becomes a problem in your life. For example, obsessive passion may make you skip a party with good friends because you feel you must adhere to a long-planned training session, but after the session, you regret your choice.

After the passion theory was described in general, we presented posts that focused on how the two passions are connected to different types of outcomes, both cognitive (intrapersonal conflict/balance) and affective outcomes. The material also focused on encouraging participants’ autonomy and promoting behavior relevant to the three psychological basic needs (autonomy, competence, relatedness). Throughout the intervention period, material related to the theoretical concepts was conveyed through the use of techniques from Motivational Interview (MI). Several cases demonstrate the effectual combination of SDT and MI on health behavior as there is a good conceptual overlap and complementarity between the approaches (Patrick and Williams, 2012). For example, material based on health-related recommendations was given in a non-corrective manner, acknowledging that participants may have different perspective while also supporting the participant’s autonomy and self-direction.

After these 4 weeks, the intervention group and control group were contacted by email to answer the same measures as the first questionnaire, in addition to addressing whether participants in the intervention group had used the intervention or not. The intervention is found at the address #dinmotivasjon at Instagram.com (to see the posts, please ask for permission to follow the account). Participants were asked about their adherence to the Instagram posts in the follow-up questionnaire. Those responding reading less than every third post were excluded from the analyses (*N* = 8).

### Measures

#### The Passion Scale

A six-item version of the Passion Scale (Vallerand et al., 2003; Vallerand, 2015) was used to assess participant’s harmonious and obsessive passion toward physical activity. The original scale consists of 12 items, with two six-item subscales. The Passion Scale has been shown to display high levels of validity and reliability in several domains (Marsh et al., 2013). The six-item scale used in the present study—used in order to minimize drop-out from the web questionnaire—was derived from previous Norwegian studies which included the full Passion Scale (Stenseng and Dalskau, 2010; Stenseng et al., 2011). The six items that best fitted a two-factorial solution from these analyses were used. These were, for obsessive passion: “I have almost an obsessive feeling for this activity,” “I have difficulties controlling my urge to do my activity,” and “This activity is so exciting that I sometimes lose control over it,” and for harmonious passion: “The new things that I discover with this activity allow me to appreciate it even more,” “This activity allows me to live a variety of experiences,” and “This activity is in harmony with the other activities in my life.”

The participants were asked to respond to each of the items scored on a 4-point Likert scale, ranging from (1) = *D*o *not agree at all* to (4) = *Very strongly agree*. In the intervention group, Cronbach’s alphas for harmonious passion were 0.72 at T1 and 0.78 at T2. For obsessive passion, Cronbach’s alphas were 0.83 at T1 and 0.86 at T2. In the control group, Cronbach’s alphas for harmonious passion were 0.72 at T1 and 0.74 at T2. For obsessive passion, Cronbach’s alphas were 0.83 at T1 and 0.83 at T2. Note that the response scale was shortened from its original 7-point to 4-point length in order to fit mobile phone screens, so that participants could use their mobile phones when responding to the questionnaire in line with the main platform of the study (Instagram).

#### Positive and Negative Affect

Items from the PANAS-X (Watson and Clark, 1999) were used to assess Positive affect (PA) and Negative affect (NA). Both PA and NA represent largely independent constructs ranging for low to high levels of emotional experience. Whereas high PA score reflects full concentration, high energy and pleasurable engagement, high NA refers to subjective distress and unpleasant engagement. The Norwegian version of the scale has been used in several studies (e.g., Stenseng et al., 2015; Stenseng and Phelps, 2016), with results supporting its validity. The questions were formulated with a reference to the stem “When having engaged in exercise, how often do you feel…” We included three items to measure positive affect (“happy,” “relaxed,” “joyful”) and three items to measure negative affect (“sad,” “distressed,” “ashamed”). Responses were made on a 4-point Likert scale ranging from (1) = *Never* to (4) *Always.* In the intervention group, Cronbach’s alphas for PA were 0.67 at T1 and 0.75 at T2. For NA, Cronbach’s alphas were 0.61 at T1 and 0.72 at T2. In the control group, Cronbach’s alphas for PA were 0.65 at T1 and 0.57 at T2. For NA, Cronbach’s alphas were 0.53 at T1 and 0.55 at T2. Notably, the low alphas are partly due to the broadness of emotions measured (e.g., being relaxed vs. joyful), and partly due to the few items in the measures; alphas tend to increase with the number of items included in a scale.

### Model Analyses

Structural equation modeling (SEM) has several advantages compared to simple multivariate test, such as ANOVA. For instance, in SEM, it is possible to perform analyses on several dependent variables in the same model, to combine latent factor analysis with regression analysis, and to use advanced statistics to adjust for missing values. In the present study, designed to investigate the effect of a 4-week social media intervention, the SEM model was defined with post-intervention measures auto-regressed on their respective pre-measures. In this way, the effect of an intervention can be determined by means of a dummy variable (0 = no intervention, 1 = intervention) regressed on the outcomes controlled for by the corresponding pre-intervention measures.

An important asset of structural equation modeling is the possibility to combine regression analysis with latent factor analysis. Hence, we built our model constructs from item-level data. The constructs of interest were included in the models at both time points, so that its counterpart (e.g., positive vs. negative affect) could be controlled for at its respective time point when including the intervention variable. Furthermore, to control for potential cross-lagged effects, paths were included controlling for the potential longitudinal interplay of variables.

The SEM analysis was conducted in M*plus* 7 (Muthen and Muthen, 2012). Missing values were treated according to the full information maximum likelihood procedure (FIML). In accordance with our hypotheses, two main models were tested, one in which the effect of the intervention was tested on the two passion constructs, and one in which the effect of the intervention was tested on positive and negative affect.

A two-step approach was applied to test both models. As recommended by Kline (2015), model fits of the latent factor constructs at each time point were validated. Second, if the factor structure yields satisfactory model fits, the full model including the regression paths is tested. If it improves model fits, covariances can be included (this means letting identical items at several time points correlate because of shared error variance).

Judgment of model fit was made according to recommendations of Hu and Bentler (1999, see also Marsh et al., 2004). Indicators of model fits were: the comparative fit index (CFI) and the Tucker-Lewis Index (TLI; both values preferably above or close to 0.95), and values of the root mean squared error of approximation (RMSEA) and the standardized root mean squared residual (SRMR; preferably less than 0.06 and 0.08, respectively). Assessment of normality of the frequency distributions in the model was conducted in light of recommendations from Lei and Lomax (2005).

## Results

In order to test the main hypotheses, the data were analyzed by means of *t*-tests, correlations, and structural equation modeling. In the following section, the main features of the data will be described, and then the correlation matrix of the variables will be inspected, and finally, the SEM analyses are presented.

### Descriptive Statistics

Mean levels and standard deviations of study variables are outlined in Table 1. The intervention group and the control group were not significantly different on all variables except age (*p* < 0.001) and gender (*p* = 0.001), as demonstrated through the *t*-tests also presented in Table 1 (independent samples *t*-test, unequal variance assumed). Based on this, age and gender were included as control variables in the later SEM analyses. Only one of the variables related to activity engagement was significantly different for the two groups, namely positive emotions at T2 (*p* = 0.05). The level of reported positive emotions related to activity engagement in the intervention group was significantly higher than for the control group, indicating that the intervention had affected the participants in the intended direction.

**Table 1:** Descriptive statistic and t-tests for variables based on groups.

	Intervention group			Control group			T-test	Significance	Cohens d
	*n*	*M*	SD	*n*	*M*	SD	*t*	*p*	*d*
Gender	226	1.04	0.19	292	1.21	0.40	6.13	<0.001	0.54
Age	226	27.17	6.68	291	25.44	4.91	3.27	0.001	0.29
Time on activity per week	225	3.4 h	0.89	292	3.6 h	0.93	0.99	0.24	0.21
T1: Harmonious passion	224	8.68	2.32	292	8.91	2.19	1.13	0.26	0.10
T2: Obsessive passion	224	5.10	2.30	289	4.93	2.25	0.85	0.40	0.07
T1: Positive emotions	224	9.54	1.30	289	9.57	1.20	0.34	0.74	0.02
T1: Negative emotions	224	4.61	1.13	289	4.56	1.00	0.54	0.59	0.04
T2: Harmonious passion	113	8.79	2.26	163	8.67	2.17	0.45	0.66	0.05
T2: Obsessive passion	113	4.92	2.31	162	4.70	1.98	0.83	0.42	0.10
T2: Positive emotions	113	9.72	1.26	162	9.43	1.09	2.00	0.046	0.25
T2: Negative emotions	112	4.61	1.29	161	4.54	1.04	0.51	0.61	0.06

At follow-up, 82% of the participants in the intervention group stated that they had read the posts for all 4 weeks, 12.4% of the participants had read the posts for approximately 3 weeks, whereas 5.4% had read the posts for 2 weeks or less. Notable, this measure was unrelated to other study variables.

### Correlations

Age and gender were modestly related to group, as also described above. However, time spent on the activity was unrelated to group, and modestly, or unrelated to age and gender at T1 and T2. The two passion constructs were correlated at T1 (*r* = 0.40) and at T2 (*r* = 0.39), and the stability between T1 and T2 ranged from 0.66 for HP and 0.75 for OP. Positive and negative affective outcomes related to the activity were negatively correlated cross-sectionally at T1 (*r* = −0.38) and T2 (*r* = −0.39). The longitudinal stability of the constructs was 0.66 (*p* < 0.001) for positive emotions and 0.91 (*p* < 0.001) for negative emotions. Correlations among the study’s variables are presented in Table 2.

**Table 2:** Bivariate correlations for study variables.

Study variables	1	2	3	4	5	6	7	8	9	10	11	12	13
1.Group	1												
2. Gender	0.258^**^	1											
3. Age	0.177^**^	0.034	1										
4. T1: Hours, activity per week	0.042	0.110^**^	0.116^**^	1									
5. T2: Hours, activity per week	−0.042	0.076	0.068	0.806^**^	1								
6. T1: Harmonious Passion	0.060	−0.057	−0.013	0.408^**^	0.381^**^	1							
7. T1: Obsessive Passion	−0.036	0.083	−0.095^*^	0.481^**^	0.509^**^	0.399^**^	1						
8. T1: Positive Emotions	0.010	−0.064	−0.049	0.162^**^	0.141^*^	0.294^**^	0.142^**^	1					
9. T1: Negative Emotions	−0.011	−0.027	0.149^**^	−0.009	0.032	−0.074	0.065	−0.380^**^	1				
10. T2: Harmonious Passion	−0.033	−0.013	−0.016	0.382^**^	0.412^**^	0.657^**^	0.353^**^	0.307^**^	0.057	1			
11. T2: Obsessive Passion	−0.066	−0.001	0.104	0.524^**^	0.522^**^	0.365^**^	0.750^**^	0.109	0.198^**^	0.391^**^	1		
12. T2: Positive Emotions	−0.114^*^	−0.118^*^	−0.066	0.100	0.142^*^	0.296^**^	0.101	0.656^**^	−0.427^**^	0.324^**^	0.138^*^	1	
13. T2: Negative Emotions	−0.021	−0.123^*^	0.195^**^	0.008	0.031	−0.087	0.084	−0.339^**^	0.901^**^	0.027	0.216^**^	−0.386^**^	1

*N = 558*.

* *p < 0.05*;

** *p < 0.01*.

### Structural Equation Modeling

Variables were inspected with regard to normality, showing values for skewness and kurtosis within conventional thresholds for normality in structural equation analyses. Only obsessive passion at T1 (skewness = 1.204) and T2 (1.115) had values >1.0 (see Lei and Lomax, 2005).

**Figure 2:**
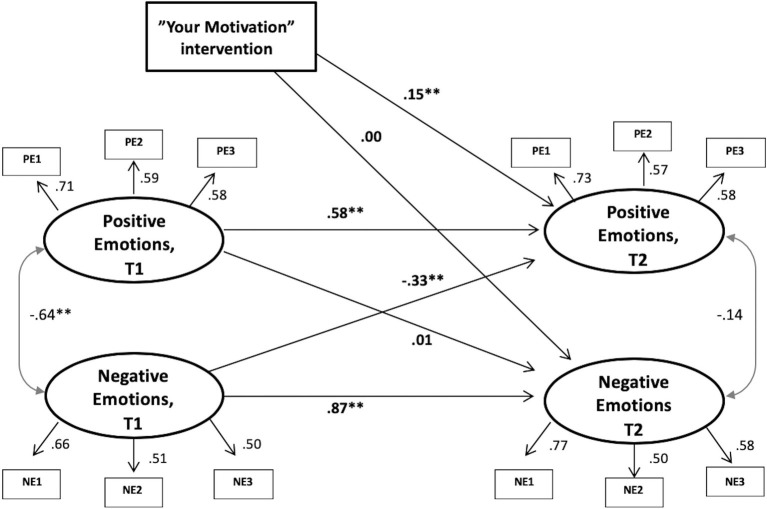
Structural equation modeling of the results. Positive and negative emotion constructs and the intervention variable. Note: **p < 0.01. Intervention variable: Intervention group = 1, Control group = 0.

In line with our hypotheses, two models were tested. The first model comprised the two passion constructs and the intervention variable (see Figure 2). In line with Kline (2015), the measurement model, which included the factor structures of the model at each time point, was first validated. These structures showed good fit with the data at both T1 [*χ*^2^(8, *N* = 518) = 22.22, *p* = 0.005, CFI = 0.98, TLI = 0.97, RMSEA = 0.059, SRMR = 0.031] and T2 [*χ*^2^(8, *N* = 277) = 8.55, *p* < 0.015, CFI = 0.99, TLI = 0.99, RMSEA = 0.016, SRMR = 0.026]. The full model, with auto-regressed paths, cross-lagged paths, and cross time covariates, showed good model fits: [*χ*^2^(52, *N* = 518) = 62.17, *p* = 0.015, CFI = 0.99, TLI = 0.99, RMSEA = 0.019, SRMR = 0.033]. Inspection of the paths in the model showed stability in both obsessive (*β* = 0.80, *p* < 0.001) and harmonious passion (*β* = 0.81, *p* < 0.001) from T1 to T2. Most importantly, the path from the variable representing assignment to the intervention had no significant effect on neither harmonious (*β* = 0.01, *p* = 0.90), nor obsessive passion (*β* = 0.00, *p* = 0.94) at T2, controlling for T1 levels. The same model was run with age and gender as control variables, but this did not lead to any substantial change of results (intervention → harmonious passion: *β* = 0.03, *p* = 0.61; intervention → obsessive passion: *β* = −0.05, *p* = 0.29).

The second model comprised the positive and negative emotion constructs as well as the intervention variable (see Appendix). This model was built in the same manner as the first, with autoregression, cross-lagged effects, and cross time covariates. The factor structure showed adequate model fits, although several items had factor loadings below 0.60, but none below 0.50: [T1 = *χ*^2^(8, *N* = 517) = 16.58, *p* = 0.034, CFI = 0.98, TLI = 0.96, RMSEA = 0.046, SRMR = 0.025; T2 = *χ*^2^(8, *N* = 277) = 14.10, *p* = 08, CFI = 0.98, TLI = 0.96, RMSEA = 0.052, SRMR = 0.034]. The full model had good fit with the data: [*χ*^2^(52, *N* = 518) = 67.89, *p* < 0.006, CFI = 0.99, TLI = 0.98, RMSEA = 0.024, SRMR = 0.033]. Inspections of the paths in the model showed that the intervention had no effect on negative emotions (*β* = 0.00, *p* = 0.97); however, the intervention had a significant positive effect on positive emotions (*β* = 0.15, *p* = 0.006). Finally, because both age and gender were significantly different in the two groups, the same model was run with those variables as control variables, without introducing any substantial change (*β* = 0.16, *p* = 0.005). Also, the intervention had no effect on time spent on the activity, controlled for T1 engagement (*β* = −0.04, *p* = 0.187).

A model with all four passion and emotion constructs in the same model was also run, in order to control for their potential overlapping relationships, but the effect of the intervention remained significant and was not substantially changed (*β* = 0.13, *p* = 0.013). In sum, the intervention had no effect on the passion constructs, nor negative emotions, but the intervention promoted positive emotions related to exercise activity engagement.

## Discussion

In the present study, we explored the effect of a social media intervention on motivation and affect related to exercise activity engagement. The intervention was designed to promote volitional engagement, harmonious passion, and positive emotions in participants’ favorite exercise activity, and to decrease contingent and extrinsic factors related to the activity, in line with self-determination theory and the dualistic model of passion (Deci and Ryan, 1985; Vallerand et al., 2003). The intervention, delivered through an Instagram account for 4 weeks, was designed to increase the participant’s knowledge and awareness of their own motivational drive through texts and images on an Instagram account. It was hypothesized that the intervention would promote harmonious passion and attenuate obsessive passion. It was also hypothesized that the intervention would affect emotions, by strengthening participant’s positive emotions and by decreasing their negative emotions. Results did not support the hypothesis that the intervention could affect participant’s passion. However, results did support the hypothesis that the intervention would strengthen positive emotions, by showing a significant change in the intervention group that was not present in the control group. Notably, more than 80% of the participants in the intervention read the posts regularly all 4 weeks, and the main findings remained when we run a more complex model with all four measures at both time points, and also when controlling for gender.

The present findings have a number of implications for the passion model. According to the DMP, an individual’s passion is self-defining and represents central features of one’s identity (Vallerand et al., 2003). Self and identity have been argued to be rather stable entities, but somewhat context sensitive (Oyserman et al., 2012). With regard to passion, experimental studies have shown that harmonious and obsessive passion may be manipulated within short time spans (Bélanger et al., 2013). However, few studies have empirically tested the stability and/or change in harmonious and obsessive passion over time in natural contexts. Apart from some exceptions (e.g., Mageau et al., 2009), harmonious passion and obsessive passion have typically been included as predictors in longitudinal research designs, and not as outcomes (e.g., Vallerand et al., 2003; Philippe et al., 2009). In consequence, the empirical body of research illuminating the stability, or whether passion can be affected, is rather sparse. Of relevance is Schellenberg and Bailis (2015) study on trajectories of academic passion among first-year university students, finding that the two passions were very stable in the overall sample (all *r*’s were > 0.62 for T1, T2, T3 data). Likewise, in a sample of teachers participating in a 3-month longitudinal study on passion and burnout-related factors, Lavigne et al. (2014) demonstrated that the test-retest correlations for the two passions were above 0.80. Also, in a work setting, Forest et al. (2012) showed that harmonious passion changed very little over a 2-month period (*r* = 0.73). The present study—showing that a social media intervention program was not able to affect levels of either harmonious or obsessive passion—adds more empirical evidence to the notion that passions are part of one’s identity and not necessarily easily amendable through low-intensity interventions such as the one used herein. In light of studies finding overlaps between obsessive passion and addiction-like engagement in activities (Vallerand et al., 2003; Stenseng et al., 2011, 2015), this indicates that more powerful interventions are needed to adjust such type of problematic engagement in exercise. Furthermore, the present study illustrates that this also counts for harmonious passion. In order to promote harmonious passion toward exercise activities in the public, more intense interventions and personal guidance may be needed to stimulate this type of adaptive motivational energy.

The Instagram intervention was also targeted toward promoting positive emotions related to exercise activity engagement. As previously shown, vigorous physical activity is related to more positive emotions in general (Steptoe and Butler, 1996) and more positive emotions are related to higher participation and better regulation of the engagement (for a review, see Vallerand and Blanchard, 2000). In other words, one route to engage individuals to participate in exercise activities is to facilitate positive emotional outcomes in the activity (Fredrickson, 2001). The Instagram intervention—through its focus on volitional engagement and psychological need satisfaction—succeeded in stimulating higher levels of positive affect from the activity. One possible explanation for this facilitating effect on affect—despite the absence of effect on the passion constructs—may be that psychological need satisfaction in activity engagement is more modifiable than passion, at least in a short period of time. While passion is seen as internalized into one’s identity, psychological need satisfaction is regarded as a more dynamic concept, dependent on both person *and* context *in present* (Deci and Ryan, 2000). Because the intervention communicated information on the satisfaction of autonomy, competence, and relatedness in a simple and straightforward manner, and related directly to exercise activity engagement, participants’ acknowledgment of such needs may have increased. More awareness of such need satisfaction may indirectly have led to more appreciation of such satisfaction in the activity, and thereby to more positive affect. Also, the intervention included images that displayed positive aspects of exercise activities. This alone may not promote positive emotions from such activity engagement, but together with the inspirational text, the images may have played a significant role in the participants’ increase in positive affect. Future intervention studies should try to differentiate the *core components* (Heath et al., 2012) of social media interventions, related to texts, figures, and clips. Furthermore, more knowledge is needed to test the ideal frequency and extent of such posts in social media interventions.

No study is without limitations, which also counts for the present one. First, participants reported an average time spent on physical activity to be 3–4 h per week, indicating that these participants’ interest was past the phase of developing a dominant type of passion. The intervention may have had a more evident effect in a sample of sedentary participants. Second, the intervention was not individualized for each participant, as the purpose of the intervention was to reach out to a wide audience. It could be beneficial to intervene on more homogeneous groups or to customize the intervention related to different types of activities (e.g., runners, dancers, or fitness athletes) or to tailor the intervention on the individual level (e.g., using the participants’ names in the inspirational text). Third, a controlled randomized design was not feasible in our study, leaving open the question of whether or not extent external factors, not measured in the study, were different in the intervention and control groups. However, the groups did not significantly differ with respect to time spent on the activity per week and how long they had been participating in the activity. They differed with respect to age and gender, but *ad hoc* analyses showed that these factors did not affect the main findings. Fourth, in order to obtain adequate response rates, short versions of scales were used to measure passion and affect. This is not ideal with regard to content validity, since leaving out items can lead to measurements issues. Fifth, emotions related to the exercise engagement were measured in a retrospective fashion, which may not capture more specific affective states during activity engagement. Readers should note that affective states, emotions, and moods are entangled and sometimes difficult to separate and report adequately (Fredrickson, 2001). Sixth, one could question whether the increase in positive emotions from the intervention is an artifact from participating in the intervention, and thus be a type of placebo effect, or results from the content in the intervention. Finally, given that the participants were recruited through social media, they seemingly are consumers of several sources of social media and thereby may have been influenced by several other channels, and may also not be representative of the total population.

## Conclusion

In conclusion, the current study extends previous research (Ryan and Deci, 2007; Vallerand, 2015) by experimentally testing the effects of social media intervention on physical exercise, under the framework of self-determination theory (Deci and Ryan, 1985, 2000) and the dualistic model of passion (Vallerand et al., 2003; Vallerand, 2015). These findings are perhaps the first to indicate that a low-intensive social media intervention is able to affect people’s positive emotions related to their training activities. Of course, given the limitations in our study, further investigations need to be conducted to verify these outcomes. Nevertheless, the present study will hopefully lead to further research on low-budget and wide-reaching interventions. As found in the present study, social media interventions can be applied to promote positive aspects of physical activity, distributed through a now common part of people’s daily life.

## Data Availability Statement

The datasets generated for this study are available on request to the corresponding author.

## Ethics Statement

The studies involving human participants were reviewed and approved by REK, Midt-Norge. The patients/participants provided their written informed consent to participate in this study.

## Author Contributions

SB planned and conducted the intervention study, partly based on previous work by JF, with supervision by FS. SB and FS conducted the analyses, in coordination with JF. SB, FS, and JF wrote the manuscript.

### Conflict of Interest

The authors declare that the research was conducted in the absence of any commercial or financial relationships that could be construed as a potential conflict of interest.

## Footnotes

^1^www.questback.com

^2^www.etikkom.no
