# Supplementary figures and images for: When Passion Does Not Change, but Emotions Do: Testing a Social Media Intervention Related to Exercise Activity Engagement
Source: Front Psychol. 2020 Feb 5;11:71. doi: 10.3389/fpsyg.2020.00071 (PMC7014931; doi:10.3389/fpsyg.2020.00071)

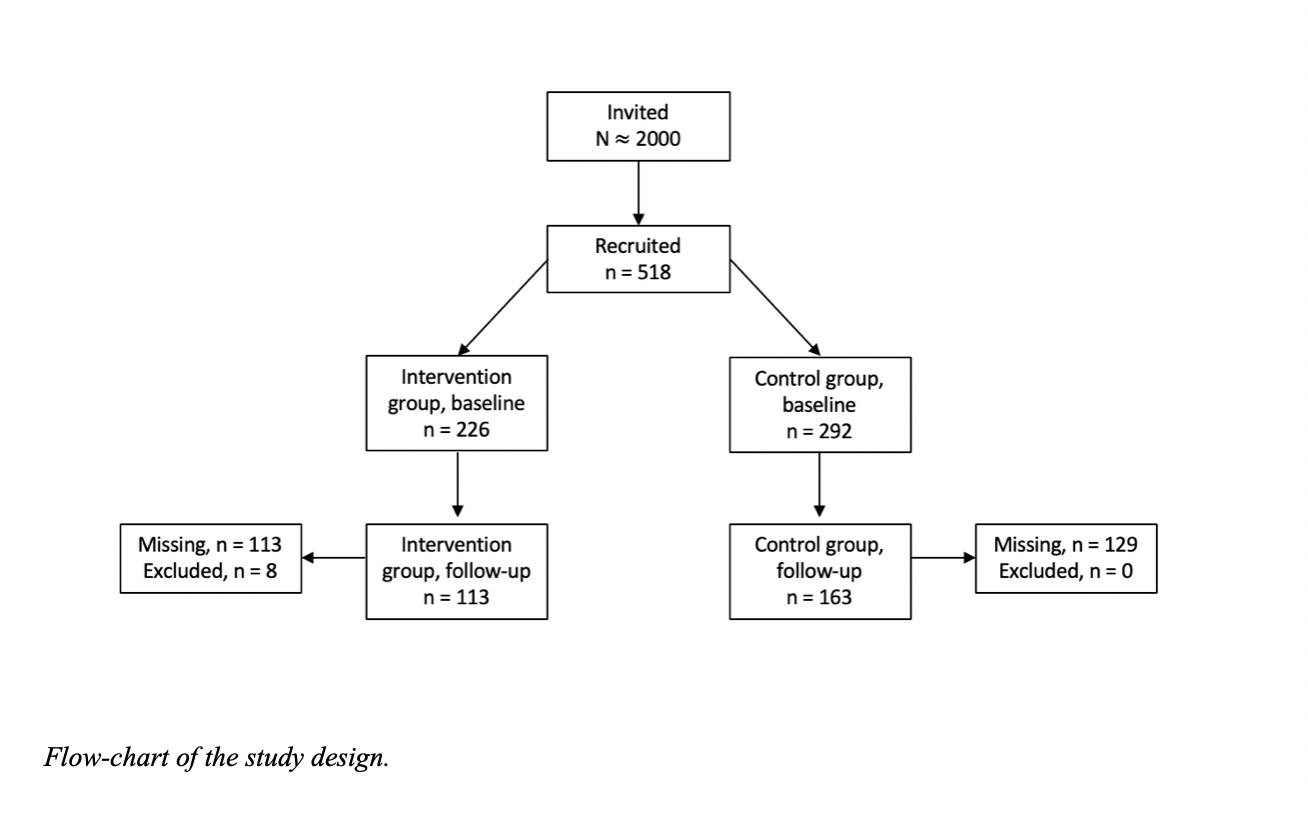

Supplement: Supplementary file 1 [file Image_1.PNG]

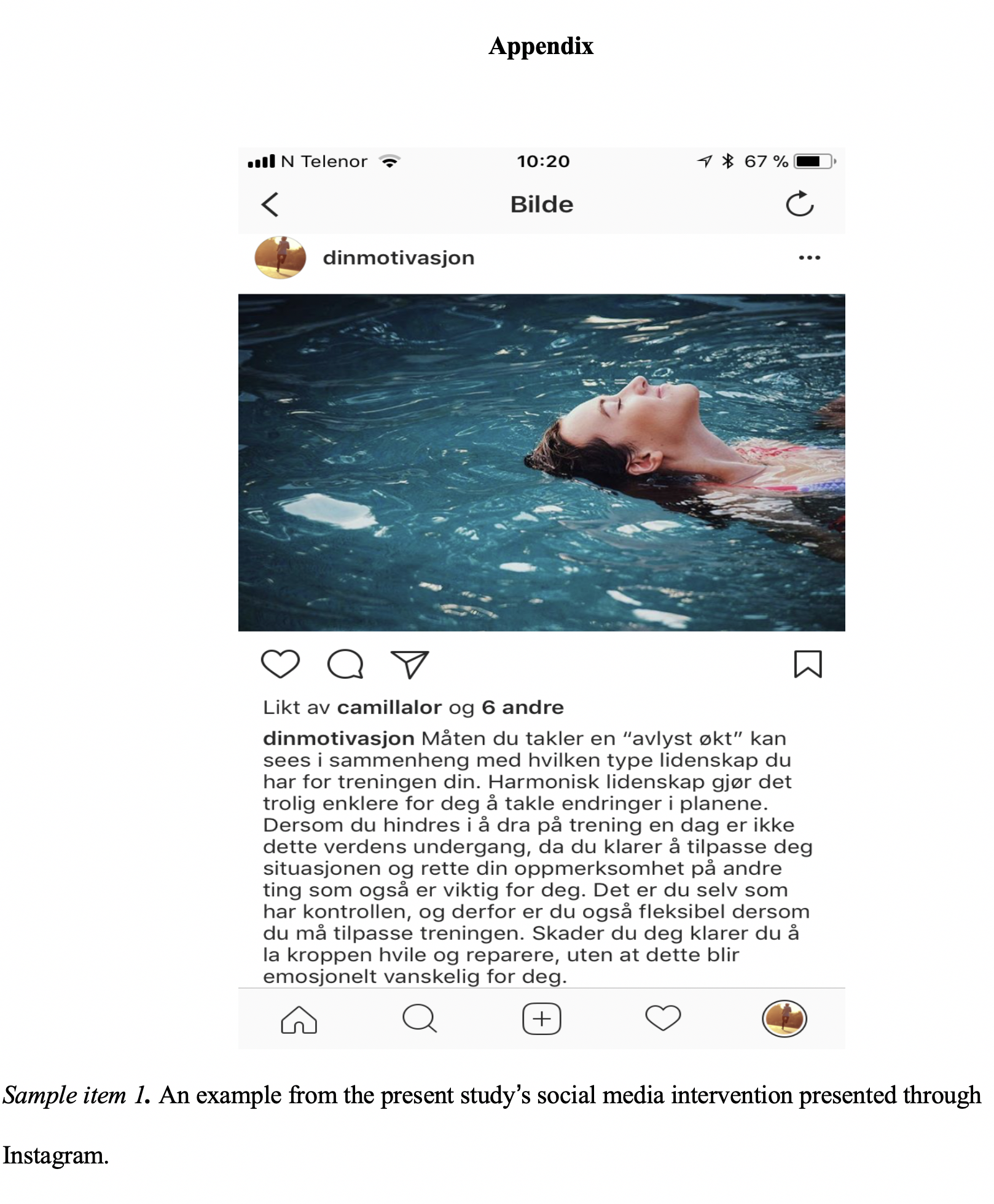

Supplement: Supplementary file 2 [file Image_2.PNG]

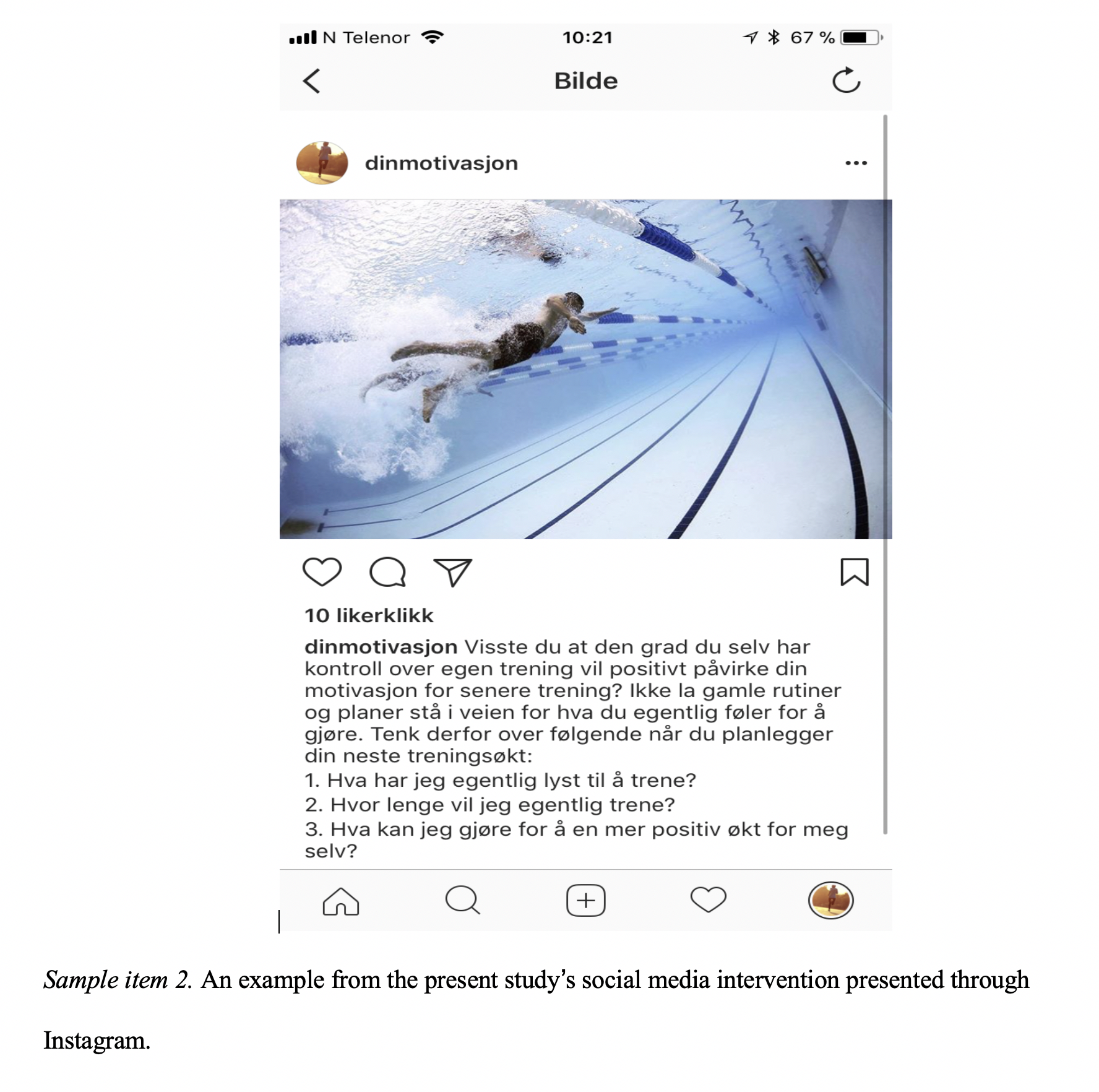

Supplement: Supplementary file 3 [file Image_3.PNG]
